# Uterine B Cells Exhibit Regulatory Properties During the Peri-Implantation Stage of Murine Pregnancy

**DOI:** 10.3389/fimmu.2019.02899

**Published:** 2019-12-11

**Authors:** Ruth Marian Guzman-Genuino, Preethi Eldi, Pablo Garcia-Valtanen, John D. Hayball, Kerrilyn R. Diener

**Affiliations:** ^1^Experimental Therapeutics Laboratory, School of Pharmacy and Medical Sciences, University of South Australia Cancer Research Institute, University of South Australia, Adelaide, SA, Australia; ^2^Adelaide Medical School, Robinson Research Institute, The University of Adelaide, Adelaide, SA, Australia

**Keywords:** uterine B cells, regulatory B cells, implantation, early pregnancy, suppression

## Abstract

A successful outcome to pregnancy is dependent on the ability of the maternal uterine microenvironment to regulate inflammation processes and establish maternal tolerance. Recently, B cells have been shown to influence pregnancy outcomes as aberrations in their numbers and functions are associated with obstetric complications. In this study, we aimed to comprehensively examine the population frequency and phenotypic profile of B cells over the course of murine pregnancy. Our results demonstrated a significant expansion in B cells within the uterus during the peri-implantation period, accompanied by alterations in B cell phenotype. Functional evaluation of uterine B cells purified from pregnant mice at day 5.5 post-coitus established their regulatory capacity as evidenced by effective suppression of proliferation and activation of syngeneic CD4^+^ T cells. Flow cytometric analysis revealed that the uterine B cell population has an expanded pool of IL-10-producing B cells bearing upregulated expression of co-stimulatory molecules CD80 and CD86 and activation marker CD27. Our investigations herein demonstrate that during the critical stages surrounding implantation, uterine B cells are amplified and phenotypically modified to act in a regulatory manner that potentially contributes toward the establishment of maternal immunological tolerance in early pregnancy.

## Introduction

Pregnancy is a model of immune tolerance wherein the mother carries a semi-allogeneic fetus that in principle triggers a maternal immune response due to the presence of paternally derived fetal alloantigens; however the inflammation is kept at a tolerable level and the pregnancy progresses. In early pregnancy, it is well-accepted that for implantation to occur, a regulated pro-inflammatory microenvironment that permits tissue remodeling and angiogenesis at the maternal-fetal interface is required. This is then followed by a shift to an immune tolerogenic profile that allows for fetal growth and development (1, 2). The necessity of a controlled inflammatory environment during this time implies a dynamic feto-maternal crosstalk of immune, endometrial, and fetal cells. A multitude of preclinical and clinical pregnancy studies show immune cells such as uterine dendritic cells, decidual macrophages, decidual natural killer cells, and regulatory T cells undergo changes during pregnancy for the purposes of calibrating inflammation and mediating maternal immune tolerance (3). For B cells, their role in the induction of maternal immune tolerance in pregnancy to date remains poorly understood.

Although studies on B cells in pregnancy are limited, there is an emerging view of their involvement in the physiology of pregnancy. Studies on murine models have demonstrated that percentages of B cell subsets are altered throughout gravidity. In the bone marrow and the spleen, murine B cells undergo lymphopenia mid-pregnancy as numbers of bone marrow pre/pro and immature B cells as well as splenic follicular B cells drop substantially at day 14 post-coitus (4). However, splenic marginal zone B cells and mature B cells in the para-aortic lymph nodes that drain the uterus were also reported to expand (4, 5). A normal pregnancy was also associated with an enhanced production of immunoglobulins by marginal zone B cells, speculated to reduce the occurrence of autoreactive B cells that may cause pregnancy complications (5). Similarly, changes in the proportions of B cell subsets during pregnancy have been documented in the clinical setting. Analysis of peripheral blood showed a significant decrease in the percentages of CD19^+^CD24^bright^CD38^bright^ transitional B cells during normal pregnancy and relatively higher percentages of non-switched memory B cells were associated with recurrent miscarriages (6, 7).

Recently, there has been interest in B cells taking on a regulatory role during pregnancy as a specific subset of B cells called “regulatory B cells” or Bregs have been implicated in the network of immune cells crucial for maintaining pregnancy. Interestingly, Bregs do not have a specific phenotypic marker, rather they are defined by their functional capacity for suppressing other immune cells (8). Among the known Breg subtypes, IL-10-producing B cells or B10 cells are the most commonly studied. Adoptive transfer of B10 cells induced from splenic B cells was previously shown to restore tolerance and rescue pregnancies in abortion-prone mice (9). Peritoneal CD19^+^IL-10^+^ cells were also shown to increase throughout normal pregnancy whereas a significant reduction was seen in abortion-prone pregnant females (10). Additionally, clinical studies correlate inadequate number of circulating IL-10-producing CD24hiCD27^+^ B cells with higher risk of spontaneous abortion (11). These studies however have been restricted to B cells in the spleen, peritoneum, and in the blood while studies of B cell function within the pregnancy microenvironment is noticeably lacking. The main hindrance to studying the B cells in the uterine mucosa and the decidua stem from the paucity of B cells within these tissues. Uterine cell profiling of the murine estrous cycle showed that B cells constituted 1.5% of viable cells at estrus (12). In humans, B cells are considered rare lymphocyte subpopulations in the uterus with a median of 2% in pre-implantation uterine tissue and 5% in the decidua (13, 14). Moreover, the functional promiscuity of B cells also poses an added layer of complexity in describing the specific role of B cells in pregnancy.

In the present work, we used a murine C57Bl/6J x BALB/c semi-allogeneic pregnancy model to describe the phenotypes and functions of B cells within the uterine microenvironment throughout gestation. Our results indicate that these uterine B cells undergo changes in frequency and phenotype, particularly during the peri-implantation phase of pregnancy between days 2.5 and 8.5 post-coitus (pc). B cells were shown to be significantly expanded as well as express markers and cytokines indicative of regulatory qualities. Furthermore, we show in *ex vivo* experiments that uterine B cells collected from pregnant females at day 5.5 pc significantly suppressed proliferation and activation of syngeneic CD4^+^ T cells via cell-cell interactions. We thus posit that uterine B cells at peri-implantation exhibit immunosuppressive characteristics and likely take part in fostering the generation of maternal immune tolerance during early pregnancy.

## Materials and Methods

### Animals

All mice were housed in a specific-pathogen free (SPF) animal facility with optimal lighting and food and water *ad libitum*. Female C57Bl/6J mice (6–9 weeks of age) were checked for estrous cycle stage by vaginal flushing with 20 μL sterile PBS and assessing the types and ratios of cells present by bright field microscopy. Females in proestrus or early estrus stages were placed with a BALB/c stud for mating overnight. The following day, female mice were visually inspected for the presence of a vaginal copulation plug indicative of a successful mating event. This was designated day 0.5 post-coitus (d0.5 pc). Virgin age-matched females at estrus were used as experimental controls.

### Tissue Dissociation and Cell Purification

At time points indicated for each experiment, the following organs were collected: spleen, the uterus draining para-aortic lymph nodes (PALNs), and uterus.

Single cell preparations from the spleen and lymph nodes were isolated by standard methods. Briefly, using the plunger of a small syringe, spleens, or lymph nodes were crushed through a 70 μm cell strainer. The resultant single cell suspension was spun (300 × g, 5 min), and resultant pellet resuspended in 1 mL of ammonium-chloride-potassium (ACK) lysis buffer for 5 min to eliminate red blood cells. RPMI culture medium supplemented with 2% fetal bovine serum (FBS) was then added to neutralize the lysis buffer. Splenocytes were washed twice with PBS, and resuspended at 10^7^ cells/mL.

Cells were extracted from uterine tissue using the gentleMACS^TM^ Octo Dissociator (Miltenyi Biotec, Bergisch Gladbach, Germany). Uterine tissue was dissected into small pieces then transferred into gentleMACS^TM^ C tubes suspended in RPMI culture medium supplemented with Collagenase A (10 mg/mL, Roche, Basel, Switzerland) and DNAse I (280 U per sample, Sigma-Aldrich, MO, USA) for efficient connective tissue digestion. Heating adaptors were used for optimal temperature control of tissue dissociation. The resultant homogenized tissue suspension was passed through a 70 μm cell strainer, resuspended in culture medium and washed twice with PBS. Collected cells were resuspended at 10^7^ cells/mL.

In experiments requiring B or T cells, the harvested mixed cells were further purified by magnetic isolation. B or T cells were negatively selected for using the MojoSort^TM^ Mouse Pan B Cell Isolation Kit (480052; Biolegend, San Diego, CA, USA) or MojoSort^TM^ Mouse CD4 T Cell Isolation Kit (480033; BioLegend) according to the manufacturer's instructions. Isolated B cells or CD4^+^ T cells were routinely achieved >90% purity as assessed by flow cytometry (Supplementary Figure 1).

For uterine tissue, the resultant cell suspension was briefly incubated with anti-mouse CD16/32 (FC block; eBioscience, San Diego, CA, USA) for 15 min at 4°C to block Fc receptors, washed, then stained with conjugated monoclonal antibody anti-mouse CD19-APC-Cy7 (BD Biosciences, NJ, USA) for 40 min in the dark on ice. CD19^+^ B cells were subjected to more restrictive FSC-W × FSC-H and SSC-W × SSC-H assessment to ensure single cells were purified by sorting on a FACS Aria II (BD Biosciences). A 100 μm nozzle at a flow rate of 2,000 events/s generated a sorting efficacy between 95 and 100%. Sorted uterine CD19^+^ B cells were used in the suppression assay described below.

### Phenotypic Analysis by Flow Cytometry

Multi-parameter flow cytometry was used to identify the expression of B cell phenotype markers in cells isolated from the organs of interest. Harvested cells were collected, washed, and then incubated with anti-mouse CD16/32 for 15 min on ice. After washing once, cells were stained with a cocktail of monoclonal antibodies diluted in buffer for 40 min. Rat/Armenian hamster anti-mouse monoclonal antibodies used are as follows: CD19-APC-Cy7 (1D3; BD Biosciences), B220-APC (RA3-6B2; BioLegend), CD1d-FITC (1B1; BD Biosciences), CD5-BV510 (53-7.3; BD Biosciences), CD38-BV421 (90/CD38; BD Biosciences), CD138-BV510 (281-2; BD Biosciences), TIM-1-BV421 (RMT 1-4; BD Biosciences), PD-L1-PE-Cy7 (MIH5; eBioscience), CD80-PE-Cy7 (16-10A1; BioLegend), CD86-AF488 (GL-1; BioLegend), CD21-BV421 (7E9; BioLegend), CD24-BV421 (M1/69; BioLegend), CD27-PE-Cy7 (LG.3A10; BioLegend), I-A/I-E-AF488 (M5/114.15.2; BioLegend), IgM-AF488 (RMM-1; BioLegend), and IgD-PE-Cy7 (11-26c.2a; BioLegend). Stained cells were washed twice with PBS then further stained with a fixable viability dye (eFluor^TM^ 660; eBioscience) for 20 min and washed with PBS prior to flow cytometric analysis (Cytoflex; Beckman Coulter, Brea, CA, USA). Post-acquisition analysis was carried out using Kaluza analysis software (Beckman Coulter). Unstained and single color controls were used to set gates and identify the positive population (Supplementary Figure 2).

Phenotypes of B cell subsets were identified as follows: CD19^+^B220^+^CD80^+^CD86^+^ activated B cells, CD19^+^B220^+^CD21^+^CD27^+^ memory B cells, CD19^+^B220^+^CD27^hi^CD38^+^CD138^+^ plasmablasts, and CD19^+^B220^+^CD24^+^IgM^hi^IgD^lo^ transitional B cells.

### *In vitro* Suppression Assay

To examine the suppressive activity of B cells collected from pregnant or virgin mice on CD4^+^ T cells, B cells from the spleen and PALNs and T cells from a syngeneic spleen were purified by magnetic isolation while B cells from the uterus were purified by FACS sorting. Purified CD4^+^ splenic T cells were labeled with Cell Proliferation Dye e450 (eBioscience) for 10 min at 37°C in the dark, then washed with 10% cold RPMI culture medium twice before resuspending in pre-warmed 10% RPMI culture medium supplemented with IL-2 (10 ng/ml, Peprotech, NJ, USA) and Dynabeads^TM^ mouse T-activator CD3/CD28 (eBioscience) for T cell expansion and activation. Cells were then dispensed into a 96-well round-bottom plate at 1 ×10^5^ cells/well, with purified B cells subsequently added to a final ratio of 0.5:1, 1:1, and 2:1 relative to T cell numbers, with unstimulated T cells and T cells with Dynabeads alone as controls. After 3 days in culture, cells were analyzed using flow cytometry to determine T cell proliferation as indicated by sequential dye dilution.

T cell proliferation was expressed as the proliferative index (PI) (15). The PI denotes the total number of divisions divided by the number of cells that went into division, and is calculated using the following formula:

$$\begin{array}{l} \text{PI}=\frac{\left(n_{pp}+\text{nG}1+\text{nG}2+\text{nG}3+\ldots \text{nGn}\right)}{\left[\left(\frac{{\text{n}}_{G1}}{2^{1}}\right)+\left(\frac{{\text{n}}_{G2}}{2^{2}}\right)+\left(\frac{{\text{n}}_{G3}}{2^{3}}\right)+\ldots \left(\frac{{\text{n}}_{Gn}}{2^{x}}\right)\right]} \end{array}$$

where *n* = number of cells in each fluorescent peak, with the peaks identified as follows: “pp” refers to the parental undivided peak of T cells, “G1” is the first T-cell division peak, “G2” the second, “G3” the third etc. until peak differentiation is not discernible from the background fluorescence. The PI for each sample was calculated using the fitting and modeling processes of the software FCS Express on cell division profiles (DeNovo Software, California, USA).

Activation of proliferating CD4^+^ cells was assessed by staining with CD4-FITC (RM4-5; eBioscience) and CD25-APC (PC61.5; eBioscience) antibodies for 40 min in the dark on ice and assessing expression by flow cytometric methods. Unstained, single-color controls, and FMOs were used as gating controls.

### Intracellular Staining of IL-10^+^ B Cells

Single cell suspensions were incubated for 5 h in a stimulation cocktail [50 ng/mL phorbol-myristate-acetate (PMA); 500 ng/mL ionomycin; 5 μg/mL lipopolysaccharide (LPS 0111:B4, Sigma)] and 1 μg/mL Brefeldin A, to induce cytokine production and inhibit Golgi transport enabling accumulation of cytokines within the cell. Cells were then washed twice and incubated with anti-mouse CD16/32 (eBioscience). Cells were washed once and stained with anti-mouse B220/CD45R-BV650 (RA3-6B2; BD Biosciences) for 40 min in the dark on ice. Post-staining, cells were fixed and permeabilized using the BD Fix/Perm Kit (BD Biosciences) as per manufacturer's instructions. Permeabilized cells were incubated with PE anti-mouse IL-10 (JES5-16E3; BioLegend) or isotype control PE Rat IgG2b κ (RTK4530; BioLegend) for 40 min at room temperature. Lastly, cells were washed twice with Perm Buffer, resuspended in FACS buffer, and analyzed for B220^+^IL-10^+^ cells via flow cytometry. Matched isotype controls were used to ensure correct gating for IL-10^+^ cells.

### Generation of Induced IL-10^+^ B Cells

Splenic B cells were magnetically isolated as above and cultured with purified anti-mouse CD40 (HM40-3; BD Biosciences) for 48 h as previously described (16). The cell culture supernatant was also collected and used for the measurement of secreted IL-10 levels by ELISA.

### IL-10 Detection by ELISA

IL-10 quantities in supernatants were measured using the ELISA Max^TM^ Standard Set Mouse IL-10 Kit (Biolegend) following the manufacturer's instructions. Briefly, high-binding 96-well plates were coated with 100 μL of the capture antibody and kept at 4°C overnight. After washing, the wells were blocked with 200 μL of 1% BSA-PBS for an hour. Samples and standards were added and incubated for 3 h, washed, and 100 μL of biotin-conjugated anti-IL-10 antibodies added. After washing, avidin-horseradish peroxidase (HRP) was added for 30 min. Wells were washed and 3,3′,5,5′-Tetramethylbenzidine (TMB) substrate solution added for 30 min for colorometric detection of IL-10. The reaction was stopped with 100 μL of 1M HCl, and plates read at 450 nm with a differential filter set at 570 nm using the MultiSkanTM FC (Thermo Fisher Scientific, USA) plate reader.

### Statistical Analysis

All experiments were performed at least in triplicate. The results were analyzed for significant differences using the Student's *t*-test or the Mann-Whitney test for parametric and non-parametric samples, respectively. For multiple comparisons, one-way ANOVA with Dunnett's *post-hoc* test was used. Differences between groups were considered statistically significant at *p* < 0.05 as analyzed using GraphPad Prism 7.03 software (GraphPad, San Diego, CA, USA).

## Results

### B Cell Proportions Are Increased in the Uterine Tissue at Peri-Implantation

To investigate the impact of pregnancy on B cell populations throughout gestation, we used a murine model of allogeneic mating where C57Bl/6J females were mated with BALB/c male studs. The percentages of B220^+^ B cells in the spleen, para-aortic (uterine draining) lymph nodes, and the uterus itself were determined at succeeding time points and compared to basal B cell numbers found in comparable organs of virgin female controls at estrus.

**Figure 1:**
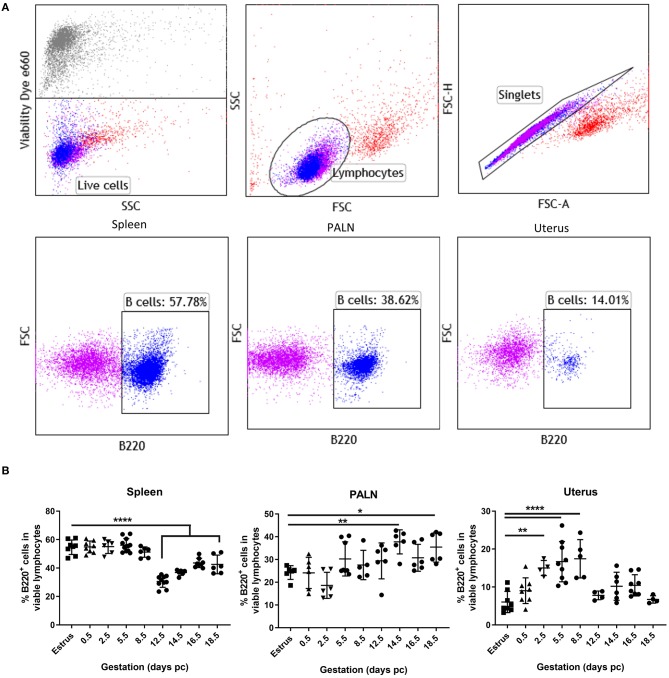
Proportions of uterine B cells expand significantly at peri-implantation. **(A)** Flow cytometric gating strategy used to identify B cells in the isolated cell suspensions from spleen, para-aortic lymph nodes, and the uterus taken at day 5.5 pc. Single viable lymphocyte cells were gated as per exclusion of the viability dye, FSCvSSC plots showing lymphocyte population, doublet discrimination, and single color controls. **(B)** Graphical summary of the percentages B220^+^ B cells in the viable lymphocyte cell populations obtained from estrus and mated mice at various days pc (*n* = 4–9 per time point). Data is shown as mean ± SEM and compared by one-way ANOVA, followed by Dunnett's *post-hoc* multiple comparisons test. *p ≤ 0.05, **p ≤ 0.01, ****p ≤ 0.0001.

Representative flow cytometric panels indicating the gating strategy and cell surface staining of viable B220^+^ B cells found within the spleen, para-aortic lymph nodes, and uterus organs at day 5.5 pc are shown in Figure 1A. Kinetic assessment of organ B cells clearly showed the induction of lymphopenia in the spleen commenced mid-pregnancy, around day 12.5 pc (Figure 1B, left panel), with a contrasting significant increase in B cells in the uterine draining lymph nodes occurring around day 14.5 pc (Figure 1B, middle panel), results that concurred with data shown in previous studies (4). In the uterus however, a statistically significant increase in B cell proportions commenced pre-implantation, at day 2.5 pc, and continued post-implantation to day 8.5 pc (Figure 1B, right panel). This increase in uterine B220^+^ B cells was limited to this peri-implantation phase as assessment of later time points revealed similar B cell frequencies to that of virgin mice in estrus. Based on these results, we focused on the day 5.5 pc time point, i.e., just after implantation, for all subsequent analyses.

### Alterations in B Cell Subsets During the Peri-Implantation Stage of Pregnancy

**Figure 2:**
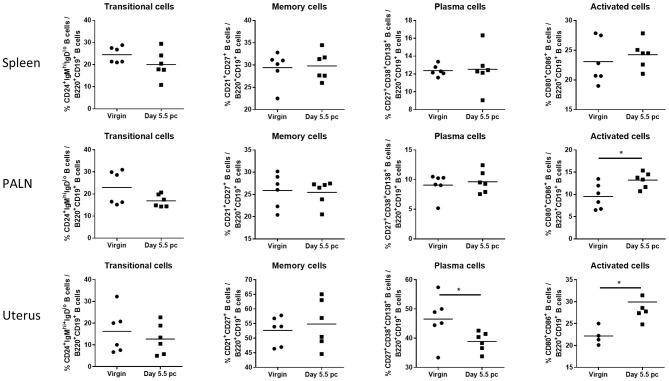
B cell subsets in the para-aortic lymph nodes and uterus are altered post-implantation. Percentages of transitional or mature naive B cells (CD24^+^IgM^hi^IgD^lo^), memory B cells (CD21^+^CD27^+^), plasma B cells (CD27^+^CD38^+^CD138^+^), and activated B cells expressing co-stimulatory molecules (CD80^+^CD86^+^) were investigated in the spleen, para-aortic (uterus draining) lymph nodes, and uterus of virgin and pregnant mice at day 5.5 pc (*n* = 6, *p*-values calculated by unpaired *t*-test). *p ≤ 0.05.

Assessment of B cell subsets from spleen, uterus draining lymph nodes, and the uterus itself at day 5.5 pc indicated that proportions of CD24^+^IgM^hi^IgD^lo^ transitional B cells and CD21^+^CD27^+^ memory B cells remained the same as those obtained from virgin female controls (Figure 2). In contrast, levels of CD27^+^CD38^+^CD138^+^ plasma B cells decreased only in the uterine tissue, with no observed changes in the spleen and the para-aortic lymph nodes. However, CD80^+^CD86^+^ activated B cells were found to be significantly higher in both the uterus and the para-aortic lymph nodes at day 5.5 pc. Together this suggests that expanded numbers of B cells at the site of implantation, and associated draining lymph nodes, are activated and upregulate co-stimulatory markers indicative of an increased capacity to interact and engage with other immune cells.

### Uterine B Cells From Pregnant Mice Suppress CD4^+^ T Cell Proliferation and Activation

Given the observed changes in B cell percentages and subset composition, we hypothesized that the upregulated expression of co-stimulatory molecules CD80 and CD86 in uterine B cells prompted a directed engagement with T cells. Potentially, co-stimulation of T cells by B cells is a pathway for T cell activation that directly affects their rate of proliferation. Thus, we sought to examine whether uterine B cells influenced CD4^+^ T cell activation and proliferation.

**Figure 3:**
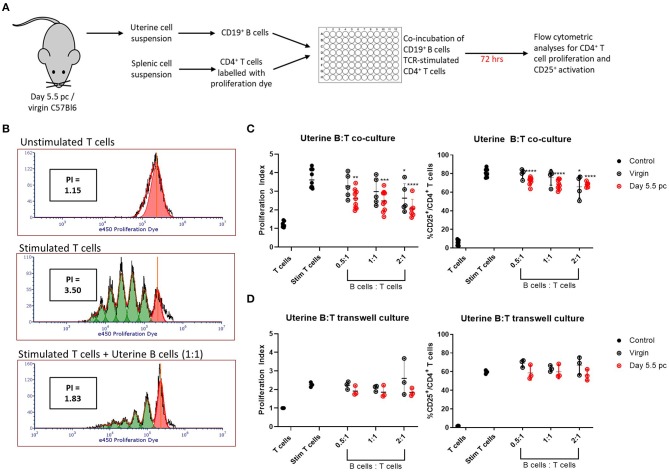
Uterine B cells from pregnant mice post-implantation suppress CD4^+^ T cell proliferation and activation. **(A)** Schematic diagram of the suppression assay used to assess the effect of B cells on CD4^+^ T cell proliferation and activation. **(B)** Representative flow cytometric panels illustrating the inhibitory effect of B cells on T cell proliferation quantified via calculation of individual proliferation index (denoted as PI). **(C)** Graphical summaries of biologically independent experiments assessing amount of CD4^+^ T cell proliferation and CD25 expression with and without co-culture of increasing ratio of uterine B cells. **(D)** The same experiments as in **(C)** except T and B cells were kept physically apart by a 0.4 μm transwell membrane (*n* = 3–8). Data is presented as mean ± SEM and compared by one-way ANOVA followed by Dunnett's *post-hoc* test. *p ≤ 0.05, **p ≤ 0.01, ***p ≤ 0.001, ****p ≤ 0.0001. Data were combined from five independent experiments.

Uteri from day 5.5 pc pregnant and virgin non-pregnant control mice were collected and processed for single cell suspensions. Viable CD19^+^ B cells sorted from the uterine cell suspensions were co-cultured with syngeneic TCR-stimulated CD4^+^ splenic T cells for 72 h, after which the T cells were assessed for cell proliferation and activation by upregulation of CD25 expression (Figure 3A). Representative histograms of T cell proliferation from unstimulated and stimulated controls, and T and B cell co-cultures are shown with corresponding proliferation index (Figure 3B). Combined results clearly indicated the ability of uterine B cells from pregnant mice to significantly inhibit the proliferation of syngeneic CD4^+^ T cells in a dose-dependent manner, with the proliferation indices significantly decreased in response to an increase in ratio of uterine B cells to T cells (Figure 3C, left panel). Similarly, inhibition of proliferation was also associated with decreased numbers of CD4^+^ T cells expressing the activation marker CD25 (Figure 3C, right panel). Furthermore, this inhibitory ability of B cells from pregnant mice was tissue-specific, as no inhibitory capacity was observed by B cells obtained from the spleen or uterus-draining lymph nodes in the same pregnant mice (Supplementary Figure 3A). B cells obtained from virgin control mice could only inhibit T cell proliferation and activation at the highest ratio of B:T cells (2:1) indicating that pregnancy amplified this basal uterine B cell response.

To determine whether the inhibitory effect was evoked by B-T cell cognate interactions or via soluble molecules produced by B cells, we conducted the same experiment but with the B cells and T cells physically separated by a 0.4 μm transwell membrane. No significant increase in inhibition of proliferation or CD25 activation was observed (Figure 3D; Supplementary Figure 3B), which suggested that the suppressive effect of uterine B cells on T cells is mediated by cognate cell-to-cell interactions rather than through soluble mediators.

### Uterine B Cells at Peri-Implantation Stage Exhibit an Enhanced Regulatory Phenotype

The previous results indicated that uterine B cells exhibited a suppressive phenotype at day 5.5 pc, post-implantation. We next sought to investigate whether these uterine B cells possessed surface and functional markers typically seen in Bregs such as the expression of TIM-1, PD-L1, CD1d, CD5, as well as the anti-inflammatory cytokine IL-10 (8). We focused particularly on investigating if there were differences in the frequencies of these known Breg phenotypes between uterine B cells from virgin mice and uterine B cells from pregnant mice that could account for their differing effects on CD4^+^ T cells.

**Figure 4:**
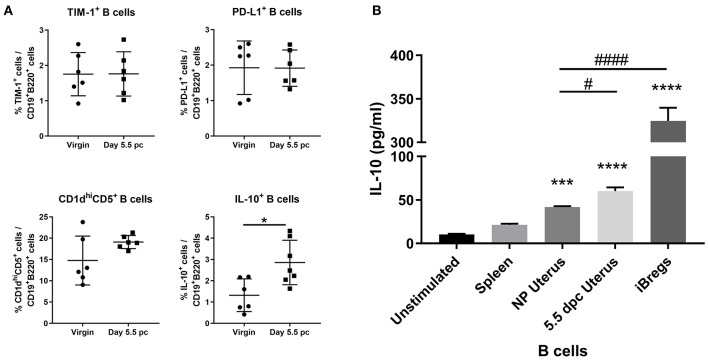
An expanded pool of IL-10^+^ B cells are present in the uterus after implantation. **(A)** Known regulatory B cell subsets were analyzed from B cells collected from non-pregnant and pregnant uteri (*n* = 6–7, unpaired *t*-test). **(B)** IL-10 production by stimulated populations of purified B cells obtained from the spleen, virgin uterus, day 5.5 pc uterus, or *in vitro* induced Bregs was assessed by ELISA. Data represent pooled supernatants of duplicate wells from three independent experiments. Data is presented as mean ± SEM and compared by one-way ANOVA, followed by Dunnett's *post-hoc* multiple comparisons test against unstimulated control. *p ≤ 0.05, ***p ≤ 0.001, ****p ≤ 0.0001. Additional Dunnett's analysis assessed against non-pregnant virgin uterus reveal a significant increase in IL-10 produced by day 5.5 pc uterine B cells and iBregs (^#^*p* ≤ 0.05, ^####^*p* ≤ 0.0001).

Assessment of surface markers indicated statistically similar proportions of TIM-1^+^ B cells, PD-L1^+^ B cells, and CD1d^hi^CD5^+^ B cells within the uterus of virgin and pregnant mice. In contrast, IL-10 cytokine expression in uterine B cells was found to be significantly increased after implantation (Figure 4A). Furthermore, *ex vivo* stimulated uterine B cells at day 5.5 pc produced levels of IL-10 that were significantly higher than unstimulated controls, stimulated splenic B cells, and stimulated uterine B cells from virgin mice, although levels are 4–5-fold less than the amount of IL-10 produced by the positive control, *in vitro* generated splenic IL-10^+^ B cells (Figure 4B). These results indicated that the capacity of uterine B cells from pregnant mice to regulate T cell proliferation and activation may be due to a significant increase in the proportion of IL-10-producing B cells within the uterine B cell population.

### Phenotype Profile of Uterine IL-10^+^ B Cells at Peri-Implantation Show Increased Activation and Co-stimulatory Markers

**Figure 5:**
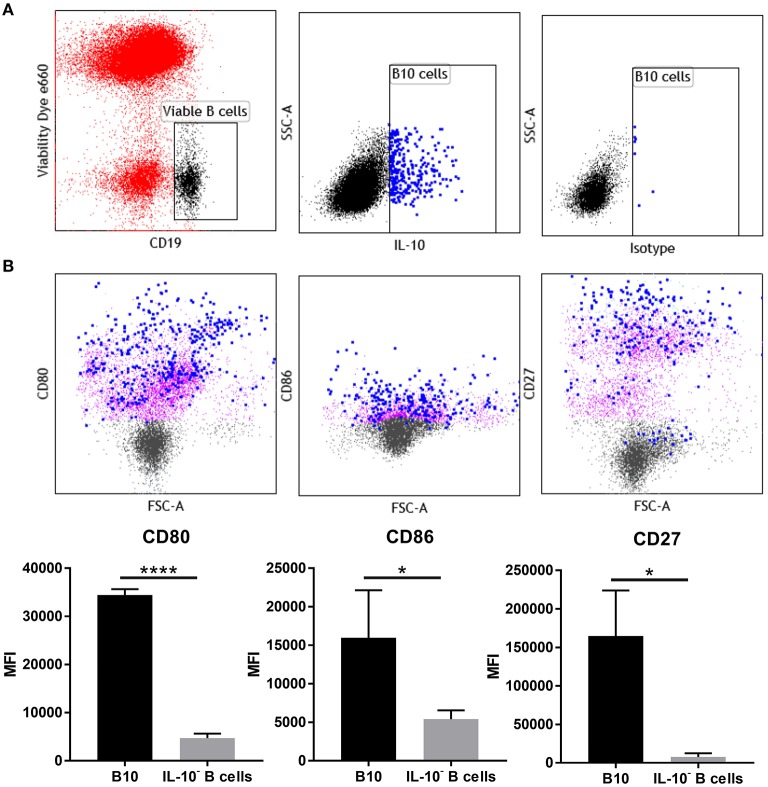
IL-10^+^ B cells at day 5.5 pc have upregulated expression of CD27, CD80, and CD86. **(A)** Gating strategy for IL-10^+^ cells within the viable CD19^+^ cell population. **(B)** Representative flow cytometric profiles of B10 cells (blue) and IL-10^−^ B cells (purple) and their upregulated expression of activation marker CD27 and co-stimulatory molecules CD80 and CD86, and the corresponding graphical presentation of the mean fluorescence intensity (MFI) of B10 cells compared to IL-10^−^ B cells by unpaired *t*-test. *p ≤ 0.05, ****p ≤ 0.0001. Data shown are from three independent experiments.

Phenotypic analysis of uterine B cells, as identified by their expression of CD19 and B220, showed that at day 5.5 pc they co-expressed two B cell receptor isotypes IgM and IgD, as well as major histocompatibility complex class II (MHC class II), signifying their antigen-driven development and maturity, as well as their capacity to act as professional antigen-presenting cells. These cells also expressed CD24, CD21, and CD38, surface markers associated with B cell differentiation and activation (Supplementary Figure 4). However, multi-parameter flow cytometric analysis of uterine IL-10^+^ B cells (Figure 5A), indicated that these cells expressed higher levels of co-stimulatory molecules CD80 and CD86 compared to non-IL-10 expressing B cells. Notably, expression of activation marker CD27 was also significantly increased in uterine IL-10^+^ B cell cells (Figure 5B). The differential expression of these markers suggests the involvement of uterine IL-10^+^ B cells in regulation of the maternal immune response at implantation, particularly through their increased capacity to engage and interact with T cells. Together with the previous findings, we posit that the enhanced expression of these co-stimulatory and activation markers accompanied with the increased production of anti-inflammatory IL-10, enable uterine B cells to regulate the proliferation and activation of CD4^+^ T cells.

## Discussion

B cells are a major component of the lymphoid immune system. Their functional promiscuity is due to the variety of B cell subsets that can carry out one or more functions such as antibody production, antigen presentation, or secretion of modulatory cytokines. Historically, B cells in pregnancy and early life have been poorly studied due to their scarcity in the pregnancy microenvironment. However, studies over the past decade have only strengthened the need to explore the role of B cells in gestation as links between B cell abnormalities and obstetric complications have been repeatedly shown (17–19).

Variations in frequency and modification in phenotype and function have been demonstrated in maternal B cells from the bone marrow and spleen during murine pregnancy, but examination of the B cell compartment within the uterine microenvironment remains limited. As the molecular architecture and function of B cells are heavily influenced by the extracellular environment and their exposure to foreign antigens (20), we hypothesized that B cells within uterine tissue in close proximity to the implanting semi-allogeneic embryo undergo changes in phenotype and function to support the development of maternal-fetal tolerance. In this study, we investigated the modifications in B cells, particularly within the uterine mucosa, in a mouse allogenic pregnancy model to provide a clearer picture of the role they play during early pregnancy.

Assessment of B cell proportions in the spleen over the gestational period recapitulate the B cell lymphopenia previously reported at mid-gestation (4). However, we also show a significant expansion in B220^+^ cells in the uterus over the peri-implantation period (days 2.5–8.5 pc; Figure 1). This expansion at the time points approaching, during, and post-implantation, suggests their involvement in the control of the inflammation required for successful decidualization and implantation. At the peri-implantation period, the morphologic and functional changes in the endometrium are accompanied by major changes in the proportion and number of endometrial immune cells (21, 22). Uterine dendritic cells, decidual macrophages, and regulatory T cells are some of the immune cells recruited into the endometrium to accumulate around the embryo at implantation (23–25). Alteration in the number and function of these cells in the local pregnancy milieu has been demonstrated to be instrumental in the recognition of fetal alloantigens, decidual differentiation, and the establishment of maternal immune tolerance (21, 22). Recently, we have shown *in vitro* that human trophoblasts can educate B cells to acquire regulatory properties that protect against T cell-mediated inflammation that may otherwise be detrimental to implantation (26). Our results herein showing the expansion of B cells in the murine pregnancy microenvironment further suggest an intimate involvement of B cells in facilitating induction of immune tolerance during the peri-implantation phase of pregnancy.

Focusing on the peri-implantation time point at day 5.5 pc (Figure 2), we showed the pregnant uterus harbors a significantly lower population of CD138^+^ plasma cells compared to unmated controls. The transmembrane marker CD138 is a syndecan specifically found in plasma cells that has long been correlated with the occurrence of chronic endometritis, an inflammatory condition of the uterine lining often associated with negative reproductive outcomes such as recurrent pregnancy loss, implantation failure, and infertility (27, 28). The decrease in the plasma cell population in the uterus, but not in the spleen or lymph nodes, gives credence to clinical evidence that plasma cell numbers should be at a tolerable level in the pregnancy microenvironment (16). Thus, the lower plasma cell count is likely suggestive of a normal implantation event.

The increased population of uterine B cells bearing CD80^+^ and CD86^+^ at day 5.5 pc is particularly interesting, as it implies increased co-stimulatory function that may direct the T cell-mediated immune response (29). Upregulation of CD80 and CD86 expression on B cells is obtained by triggering and activating the BCR through a variety of stimuli such as ligands and cytokines (30, 31). Subsequently, the crosstalk facilitated by the enhanced expression of these two co-stimulatory molecules on activated B cells is capable of skewing T cell activity (29). In pregnancy, it has been demonstrated that increased CD86 expression in murine peritoneal cavity B1-a B cells is associated with perturbations to pregnancy in a CBA/J mice model, an effect attributed to the increased capacity for Th1 and Th17 differentiation (32). It may however be a different case within the pregnancy microenvironment as investigated here, where the presence of the blastocyst and associated alterations in cytokine production within the microenvironment may serve as triggers for BCR activation and lead to enhanced expression of both CD86 and CD80 on uterine B cells. Moreover, previous studies demonstrated the upregulated expression of CD80 and CD86 on murine splenic antigen presenting cells such as dendritic cells and macrophages at the pre-implantation period (day 3.5 pc). This was shown to be crucial in modulating T regulatory cell (Treg) abundance, cytokine production, and ultimately pregnancy outcome (33, 34). A follow up study examining splenic B cells showed similar results, with CD86 protein expression significantly increased in normal pregnant mice compared to abortion-prone models at pre-implantation (35). Thus, we postulate that the differential expression of CD80 and CD86 in uterine B cells may be predisposing them to act in a similar manner, that is elevated expression of these molecules on uterine B cells may contribute to establishment of maternal tolerance and therefore appropriate implantation. These findings are certainly intriguing and warrant further investigation that was unfortunately, out of scope of this current study.

Amongst the B cell subsets, regulatory B cells or Bregs have drawn attention as potentially critical players in pregnancy well-being. Previous murine studies have shown that splenic Bregs can rescue pregnancies in abortion-prone models (9), and a clinical study has revealed that deficiency in circulating IL-10-producing Bregs is correlated with higher risk of spontaneous abortions (11). Furthermore, it was recently shown that the lack of IL-10^+^ B cells in murine pregnancies led to diminished embryo sizes and stagnated Treg pools, as well as increased susceptibility to LPS which caused intrauterine fetal death (36). With the expansion of uterine B cells at the peri-implantation phase, we speculated whether these cells possessed regulatory properties, and indeed demonstrated that uterine B cells from day 5.5 pc pregnant mice effectively suppressed syngeneic CD4^+^ T cell proliferation and activation as seen by the significant reduction in proliferation and diminished expression of the activation marker CD25 in a cell contact dependent manner (Figure 3).

Bregs are defined as any B cell carrying out negative regulation of an immune response and as such, a plethora of studies have attributed this ability to various subsets of B cells including TIM-1^+^ B cells, PD-L1^+^ B cells, CD5^+^CD1d^hi^ B cells, and IL-10^+^ B cells (8). Among these subsets, only the IL-10^+^ B cells were significantly expanded in the uterus at the peri-implantation stage (day 5.5 pc) of pregnancy, with more IL-10 produced by B cells compared to non-pregnant controls and splenic B cells (Figure 4). In pregnancy, IL-10 is found in high amounts in placental and decidual tissues at early gestation in pre-clinical and clinical studies and is well-established as a vital cytokine for optimal pregnancy outcomes (37). Mainly thought to be produced by trophoblasts, uterine natural killer cells, monocytes, dendritic cells, and Tregs, IL-10 has been shown to be a key cytokine for the balance of pro- and anti-inflammatory signals that establish maternal immune tolerance during the implantation process (38–40). While it was desirable to assess the suppressive capacity of these uterine IL-10^+^ B cells on CD4^+^ T cells specifically, the low percentage of IL-10^+^ B cells (2–5%) within an already scarce population of uterine B cells from an individual pregnant mouse proved to be a major limitation to conducting these experiments.

**Figure 6:**
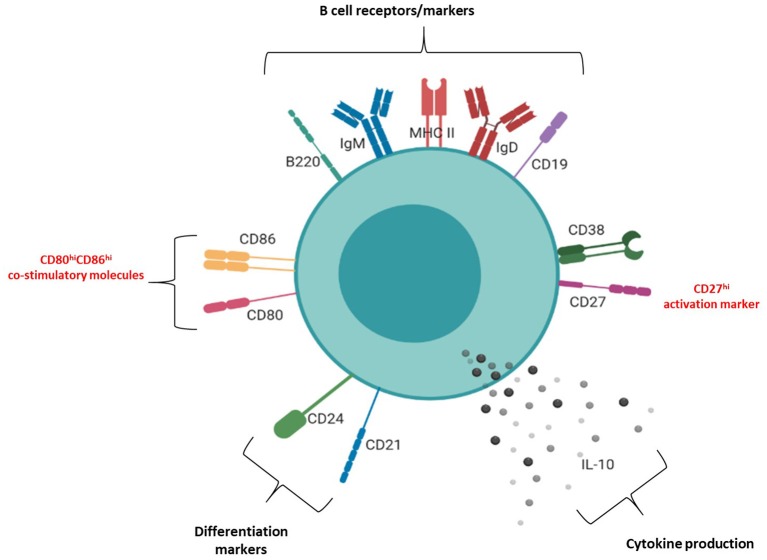
Phenotypic structure of an IL-10^+^ uterine B cell at peri-implantation. Schematic diagram of the phenotypic markers expressed by uterine IL-10^+^ B cells at peri-implantation, including B cell receptors and markers (CD19, B220, IgM, IgD, MHC II), activation markers (CD27, CD38), differentiation markers (CD21, CD24), co-stimulatory molecules (CD80, CD86), and IL-10 cytokine production.

The molecular surface architecture of uterine IL-10^+^ B cells is summarized in Figure 6 and illustrates how these cells may facilitate their suppressive function. We determined that the majority of uterine B cells isolated from pregnant mice at the peri-implantation time point take on a phenotype descriptive of mature and antigen-driven differentiated B cells: they bear both IgM and IgD, antigen presenting cell marker MHC class II, differentiation markers CD24 and CD21, and activation marker CD38 (Supplementary Figure 4). The dual expression of IgM and IgD is particularly interesting, as IgD co-expression is suggested to indicate a “tolerogenic” role via regulating autoreactivity brought about by chronic exposure to antigen (6). Likewise, the retention of differentiation markers CD24 and CD21 as well as expression of activation marker CD38 suggested that uterine B cells are mature, but yet to have differentiated into antibody-producing plasma cells (41, 42). Furthermore, ~20 and 50% of IL-10 negative uterine B cells express the ligands CD80 and CD86, respectively (Figure 5B) which suggests that a significant proportion of the uterine B cells have the capacity for co-stimulating T cell activation, potentially leading to negative regulation by way of Treg expansion and modification of cytokine production (33, 34). Interestingly, the small proportion observed as IL-10^+^ B cells within the uterine B cell population exhibited higher expression of co-stimulatory molecules CD80 and CD86 as well as CD27, an activation marker of memory and plasmablast lineages in murine B cells. In this study, these cell surface molecules, together with the cells' capacity for producing anti-inflammatory IL-10, is likely to have contributed to the regulatory phenotype that uterine B cells from pregnant mice exhibit. The differential expression of these surface markers indicates that IL-10^+^ B cells may be involved in facilitating changes in the maternal immune network as it adjusts to the presence of fetal allo-antigens in early pregnancy. The increased capacity and engagement of these activated IL-10^+^ B cells for T cell co-stimulation potentially contributes to the range of the T cell repertoire generated in early pregnancy, a process critical for setting the conditions for implantation.

## Conclusion

To our knowledge, this is the first comprehensive study on the phenotype and functional characteristics of uterine B cells in early murine allogeneic pregnancy. In this study, we demonstrate an expanded pool of uterine B cells at the time points associated with embryo implantation, which harbor expanded subpopulations of CD80^+^CD86^+^ and IL-10^+^ B cells, which collectively suppresses CD4^+^ T cell proliferation and activation. Thus, we propose that at this crucial time point, B cells within the uterine microenvironment undergo changes in both population frequency and phenotype that supports a regulatory role and contributes to the immune changes necessary for the progression of a successful pregnancy.

## Data Availability Statement

The raw data that support the findings of this study are available from the corresponding authors upon reasonable request.

## Ethics Statement

The animal study was reviewed and approved by University of South Australia Animal Ethics Committee. All animal work was conducted in accordance with the Australian Code for the Care and Use of Animals for Scientific Purposes 8th edition (2013) as defined by the National Health and Medical Research Council of Australia. All experiments were conducted with approval from The University of Adelaide and University of South Australia Animal Ethics Committees.

## Author Contributions

KD and RG-G conceived and designed the study. RG-G performed the experiments, analyzed the data, and wrote the manuscript. PE, PG-V, and JH assisted in experimental design and provided technical expertise. KD evaluated the results and edited the manuscript. KD, JH, PE, and PG-V supervised the study.

### Conflict of Interest

The authors declare that the research was conducted in the absence of any commercial or financial relationships that could be construed as a potential conflict of interest.

## References

[1] Mor G Cardenas I. The immune system in pregnancy: a unique complexity. Am J Reprod Immunol. (2010) 63:425–33. 10.1111/j.1600-0897.2010.00836.x 20367629 PMC3025805

[2] Ghaebi M Nouri M Ghasemzadeh A Farzadi L Jadidi-Niaragh F Ahmadi M. Immune regulatory network in successful pregnancy and reproductive failures. Biomed Pharmacother. (2017) 88:61–73. 10.1016/j.biopha.2017.01.016 28095355

[3] Schumacher A Sharkey DJ Robertson SA Zenclussen AC. Immune cells at the fetomaternal interface: how the microenvironment modulates immune cells to foster fetal development. J Immunol. (2018) 201:325–34. 10.4049/jimmunol.1800058 29987001

[4] Muzzio DO Soldati R Ehrhardt J Utpatel K Evert M Zenclussen AC. B cell development undergoes profound modifications and adaptations during pregnancy in mice. Biol Reprod. (2014) 91:115. 10.1095/biolreprod.114.122366 25210132

[5] Muzzio DO Ziegler KB Ehrhardt J Zygmunt M Jensen F. Marginal zone B cells emerge as a critical component of pregnancy well-being. Reproduction. (2016) 151:29–37. 10.1530/REP-15-0274 26493101

[6] Noviski M Mueller JL Satterthwaite A Garrett-Sinha LA Brombacher F Zikherman J. IgM and IgD B cell receptors differentially respond to endogenous antigens and control B cell fate. Elife. (2018) 7:e35074. 10.7554/eLife.35074 29521626 PMC5897097

[7] Ziegler KB Muzzio DO Matzner F Bommer I Ventimiglia MS Malinowsky K. Human pregnancy is accompanied by modifications in B cell development and immunoglobulin profile. J Reprod Immunol. (2018) 129:40–7. 10.1016/j.jri.2018.07.003 30165265

[8] Guzman-Genuino RM Diener KR. Regulatory B cells in pregnancy: lessons from autoimmunity, graft tolerance, and cancer. Front Immunol. (2017) 8:172. 10.3389/fimmu.2017.00172 28261223 PMC5313489

[9] Jensen F Muzzio D Soldati R Fest S Zenclussen AC. Regulatory B10 cells restore pregnancy tolerance in a mouse model. Biol Reprod. (2013) 89:90. 10.1095/biolreprod.113.110791 23986569

[10] Schumacher A Ehrentraut S Scharm M Wang H Hartig R Morse HC III Plasma cell alloantigen 1 and IL-10 secretion define two distinct peritoneal B1a B cell subsets with opposite functions, PC1(high) cells being protective and PC1(low) cells harmful for the growing fetus. Front Immunol. (2018) 9:1045 10.3389/fimmu.2018.01045 29868008 PMC5962664

[11] Rolle L Memarzadeh Tehran M Morell-Garcia A Raeva Y Schumacher A Hartig R. Cutting edge: IL-10-producing regulatory B cells in early human pregnancy. Am J Reprod Immunol. (2013) 70:448–53. 10.1111/aji.12157 24118333

[12] Diener KR Robertson SA Hayball JD Lousberg EL. Multi-parameter flow cytometric analysis of uterine immune cell fluctuations over the murine estrous cycle. J Reprod Immunol. (2016) 113:61–7. 10.1016/j.jri.2015.11.005 26759962

[13] Rieger L Segerer S Bernar T Kapp M Majic M Morr AK. Specific subsets of immune cells in human decidua differ between normal pregnancy and preeclampsia–a prospective observational study. Reprod Biol Endocrinol. (2009) 7:132. 10.1186/1477-7827-7-132 19930648 PMC2789084

[14] Feyaerts D Benner M van Cranenbroek B van der Heijden OWH Joosten I van der Molen RG. Human uterine lymphocytes acquire a more experienced and tolerogenic phenotype during pregnancy. Sci Rep. (2017) 7:2884. 10.1038/s41598-017-03191-0 28588205 PMC5460245

[15] Diener KR Moldenhauer LM Lyons AB Brown MP Hayball JD. Human Flt-3 ligand-mobilized dendritic cells require additional activation to drive effective immune responses. Exp Hematol. (2008) 36:51–60. 10.1016/j.exphem.2007.08.024 17949888

[16] Matsushita T Tedder TF. Identifying regulatory B cells (B10 cells) that produce IL-10 in mice. Methods Mol Biol. (2011) 677:99–111. 10.1007/978-1-60761-869-0_7 20941605

[17] Jensen F Wallukat G Herse F Budner O El-Mousleh T Costa SD. CD19^+^CD5^+^ cells as indicators of preeclampsia. Hypertension. (2012) 59:861–8. 10.1161/HYPERTENSIONAHA.111.188276 22353610

[18] Shigeta N Nakamura H Kumasawa K Imai K Saito S Sakaguchi S Are naive T cells and class-switched memory (IgD(^−^) CD27(^+^)) B cells not essential for establishment and maintenance of pregnancy? Insights from a case of common variable immunodeficiency with pregnancy. Med Hypotheses. (2018) 121:36–41. 10.1016/j.mehy.2018.09.014 30396484

[19] Marron K Harrity C. Endometrial lymphocyte concentrations in adverse reproductive outcome populations. J Assist Reprod Genet. (2019) 36:837–46. 10.1007/s10815-019-01427-8 30847699 PMC6541687

[20] Lebien TW Tedder TF. B lymphocytes: how they develop and function. Blood. (2008) 112:1570–80. 10.1182/blood-2008-02-078071 18725575 PMC2518873

[21] Lee JY Lee M Lee SK. Role of endometrial immune cells in implantation. Clin Exp Reprod Med. (2011) 38:119–25. 10.5653/cerm.2011.38.3.119 22384430 PMC3283071

[22] Robertson SA Moldenhauer LM. Immunological determinants of implantation success. Int J Dev Biol. (2014) 58:205–17. 10.1387/ijdb.140096sr 25023687

[23] Plaks V Birnberg T Berkutzki T Sela S Benyashar A Kalchenko V. Uterine DCs are crucial for decidua formation during embryo implantation in mice. J Clin Invest. (2008) 118:3954–65. 10.1172/JCI36682 19033665 PMC2582932

[24] Nagamatsu T Schust DJ. The contribution of macrophages to normal and pathological pregnancies. Am J Reprod Immunol. (2010) 63:460–71. 10.1111/j.1600-0897.2010.00813.x 20163399

[25] Shima T Sasaki Y Itoh M Nakashima A Ishii N Sugamura K Regulatory T cells are necessary for implantation and maintenance of early pregnancy but not late pregnancy in allogeneic mice. J Reprod Immunol. (2010) 85:121–9. 10.1016/j.jri.2010.02.006 20439117

[26] Guzman-Genuino RM Dimova T You Y Aldo P Hayball JD Mor G. Trophoblasts promote induction of a regulatory phenotype in B cells that can protect against detrimental T cell-mediated inflammation. Am J Reprod Immunol. (2019) 82:e13187. 10.1111/aji.13187 31487409 PMC8232043

[27] Chen YQ Fang RL Luo YN Luo CQ. Analysis of the diagnostic value of CD138 for chronic endometritis, the risk factors for the pathogenesis of chronic endometritis and the effect of chronic endometritis on pregnancy: a cohort study. BMC Womens Health. (2016) 16:60. 10.1186/s12905-016-0341-3 27596852 PMC5477816

[28] Song D Feng X Zhang Q Xia E Xiao Y Xie W Prevalence and confounders of chronic endometritis in premenopausal women with abnormal bleeding or reproductive failure. Reprod Biomed. (2018) 36:78–83. 10.1016/j.rbmo.2017.09.008 29111313

[29] Sahoo NC Rao KV Natarajan K. CD80 expression is induced on activated B cells following stimulation by CD86. Scand J Immunol. (2002) 55:577–84. 10.1046/j.1365-3083.2002.01093.x 12028560

[30] Mongini PK Tolani S Fattah RJ Inman JK. Antigen receptor triggered upregulation of CD86 and CD80 in human B cells: augmenting role of the CD21/CD19 co-stimulatory complex and IL-4. Cell Immunol. (2002) 216:50–64. 10.1016/S0008-8749(02)00512-9 12381350

[31] Suvas S Singh V Sahdev S Vohra H Agrewala JN. Distinct role of CD80 and CD86 in the regulation of the activation of B cell and B cell lymphoma. J Biol Chem. (2002) 277:7766–75. 10.1074/jbc.M105902200 11726649

[32] Muzzio DO Soldati R Rolle L Zygmunt M Zenclussen AC Jensen F. B-1a B cells regulate T cell differentiation associated with pregnancy disturbances. Front Immunol. (2014) 5:6. 10.3389/fimmu.2014.00006 24478775 PMC3896948

[33] Slawek A Maj T Chelmonska-Soyta A. CD40, CD80, and CD86 costimulatory molecules are differentially expressed on murine splenic antigen-presenting cells during the pre-implantation period of pregnancy, and they modulate regulatory T-cell abundance, peripheral cytokine response, and pregnancy outcome. Am J Reprod Immunol. (2013) 70:116–26. 10.1111/aji.12108 23445188

[34] Maj T Slawek A Chelmonska-Soyta A. CD80 and CD86 costimulatory molecules differentially regulate OT-II CD4(^+^) T lymphocyte proliferation and cytokine response in cocultures with antigen-presenting cells derived from pregnant and pseudopregnant mice. Mediators Inflamm. (2014) 2014:769239. 10.1155/2014/769239 24771983 PMC3977523

[35] Lorek D Kedzierska AE Slawek A Chelmonska-Soyta A. Expression of Toll-like receptors and costimulatory molecules in splenic B cells in a normal and abortion-prone murine pregnancy model. Am J Reprod Immunol. (2019) 82:e13148. 10.1111/aji.13148 31134706

[36] Busse M Campe KJ Nowak D Schumacher A Plenagl S Langwisch S. IL-10 producing B cells rescue mouse fetuses from inflammation-driven fetal death and are able to modulate T cell immune responses. Sci Rep. (2019) 9:9335. 10.1038/s41598-019-45860-2 31249364 PMC6597542

[37] Thaxton JE Sharma S. Interleukin-10: a multi-faceted agent of pregnancy. Am J Reprod Immunol. (2010) 63:482–91. 10.1111/j.1600-0897.2010.00810.x 20163400 PMC3628686

[38] White CA Johansson M Roberts CT Ramsay AJ Robertson SA. Effect of interleukin-10 null mutation on maternal immune response and reproductive outcome in mice. Biol Reprod. (2004) 70:123–31. 10.1095/biolreprod.103.018754 13679317

[39] Robertson SA Care AS Skinner RJ. Interleukin 10 regulates inflammatory cytokine synthesis to protect against lipopolysaccharide-induced abortion and fetal growth restriction in mice. Biol Reprod. (2007) 76:738–48. 10.1095/biolreprod.106.056143 17215490

[40] Robertson SA Chin PY Glynn DJ Thompson JG. Peri-conceptual cytokines–setting the trajectory for embryo implantation, pregnancy and beyond. Am J Reprod Immunol. (2011) 66(Suppl 1):2–10. 10.1111/j.1600-0897.2011.01039.x 21726333

[41] Oliver AM Martin F Kearney JF. Mouse CD38 is down-regulated on germinal center B cells and mature plasma cells. J Immunol. (1997) 158:1108–15. 9013949

[42] Benitez A Weldon AJ Tatosyan L Velkuru V Lee S Milford TA. Differences in mouse and human nonmemory B cell pools. J Immunol. (2014) 192:4610–9. 10.4049/jimmunol.1300692 24719464 PMC4046845

