# Supplementary material for: Uterine B Cells Exhibit Regulatory Properties During the Peri-Implantation Stage of Murine Pregnancy
Source: Front Immunol. 2019 Dec 11;10:2899. doi: 10.3389/fimmu.2019.02899 (PMC6917594; doi:10.3389/fimmu.2019.02899)
Supplement: Supplementary file 1 [file Data_Sheet_1.pdf]

frontiers

Supplementary Material

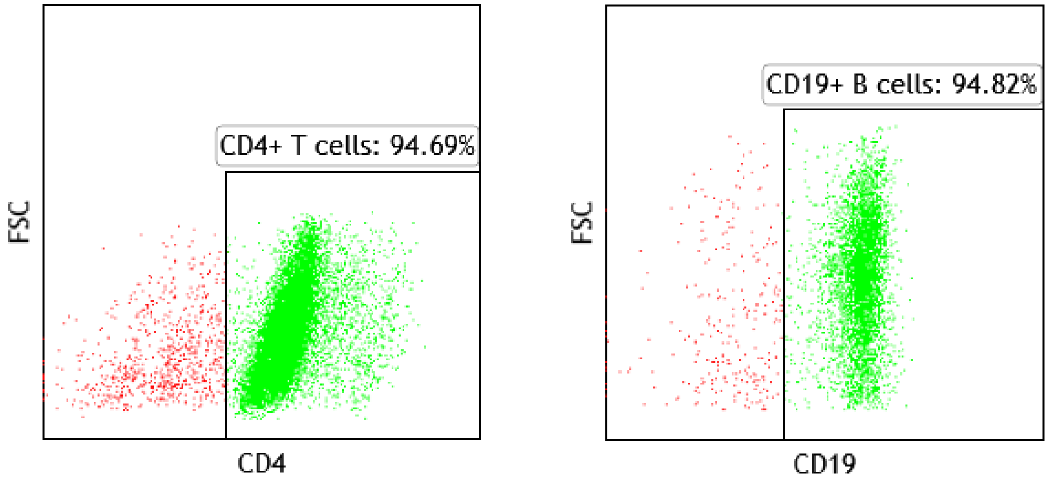

Supplementary Figure 1. Flow cytometric assessment of CD4$^+$ T cell and B cell purification methods. CD4$^+$ T cells were purified from female C57Bl/6J splenocyte single cell suspensions by negative selection via magnetic isolation. Similarly, B cells were purified from single cell preparations of spleen and the para-aortic (uterine draining) lymph nodes by negative selection while B cells from uterine tissue were purified by FACS sorting. Assessment of resultant CD4$^+$ T cell and B cell purity was performed by staining samples of purified cell preparations with anti-CD4 or anti-CD19 antibodies respectively, followed by flow cytometric analysis. Shown are representative dot plots which indicate that each purification technique resulted in >90% purity.

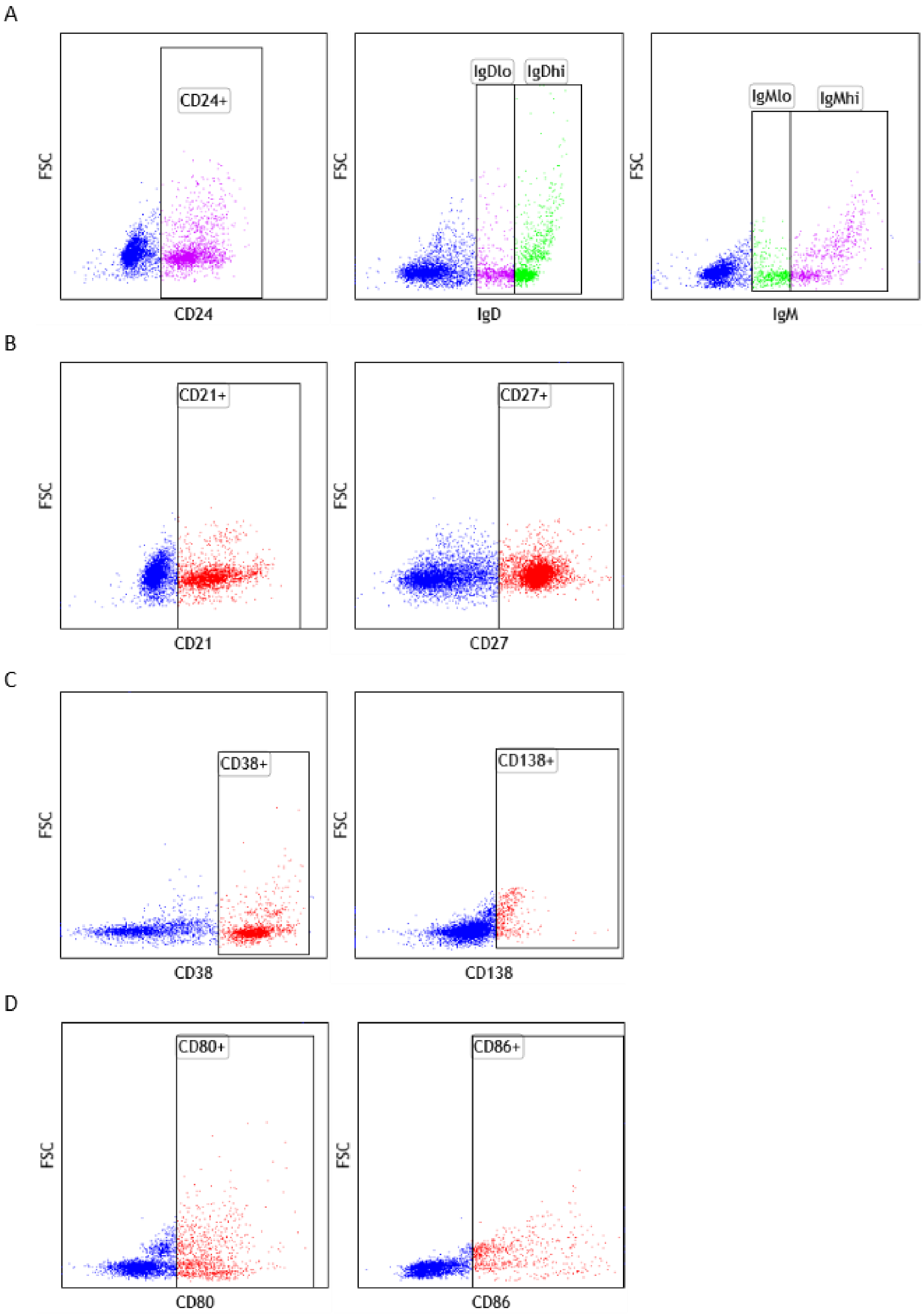

Supplementary Figure 2. Single color controls for establishing the gating strategies of various B cell populations. Single staining was used to determine the fluorescence parameters of populations positive or negative for surface marker expression. Parameters were applied to $CD19^{+}B220^{+}$ B cells for the identification of the following groups: (A) $CD24^{+}IgM^{hi}IgD^{lo}$ transitional B cells; (B) $CD21^{+}CD27^{+}$ memory B cells; $CD27^{+}CD38^{+}CD138^{+}$ plasma cells; (D) $CD80^{+}CD86^{+}$ activated B cells.

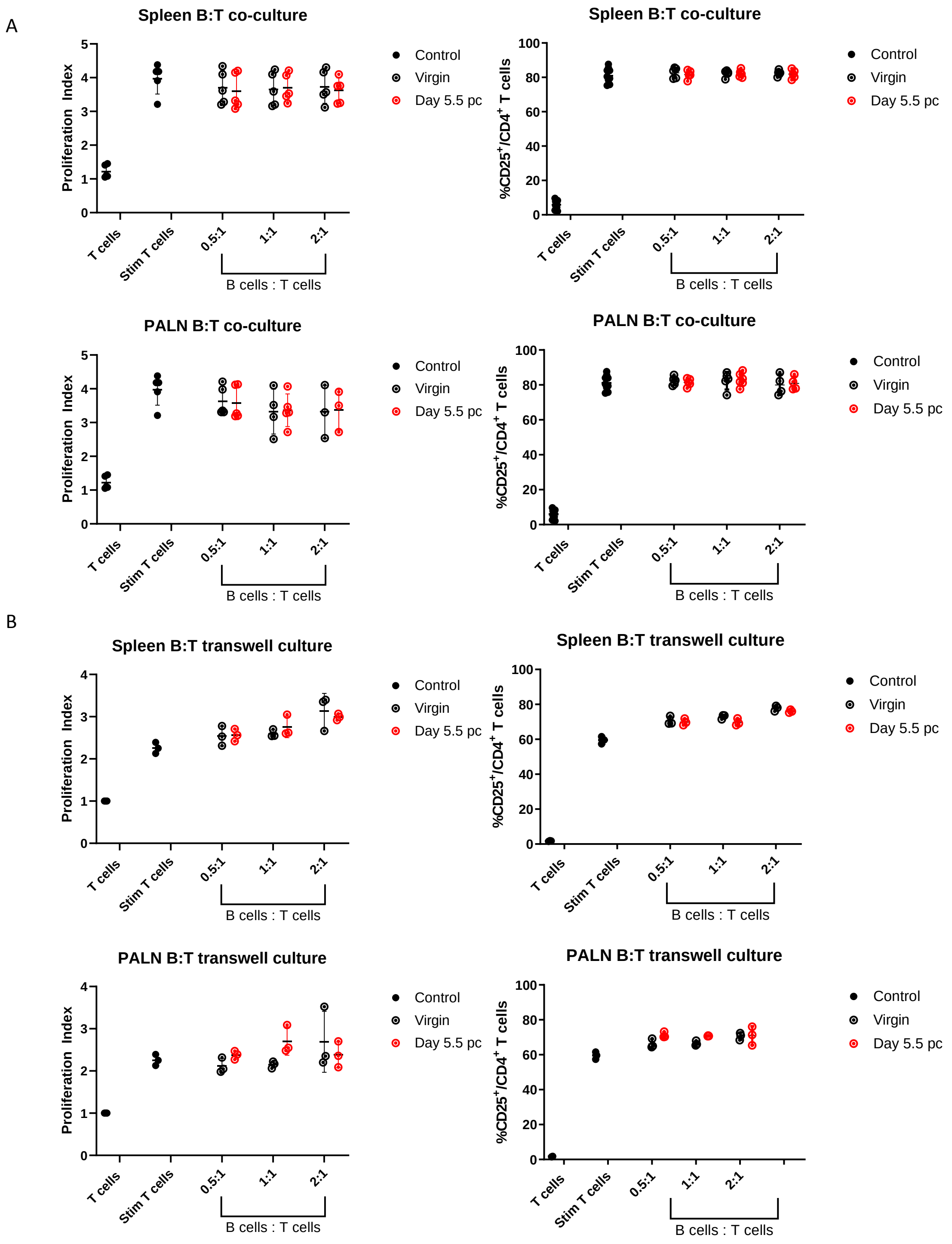

Supplementary Figure 3. (A) No statistical differences in proliferation indices of CD4$^+$ T cells incubated with increasing ratios of B cells isolated from the spleen and para-aortic lymph nodes (PALN) in a co-culture (n=5). (B) No observed differences in CD25$^+$ activation of CD4$^+$ T cells incubated with increasing ratios of B cells isolated from the spleen and para-aortic lymph nodes (PALN), separated physically via transwell membranes (n=3). Data presented from three to five independent experiments and compared using one-way ANOVA.

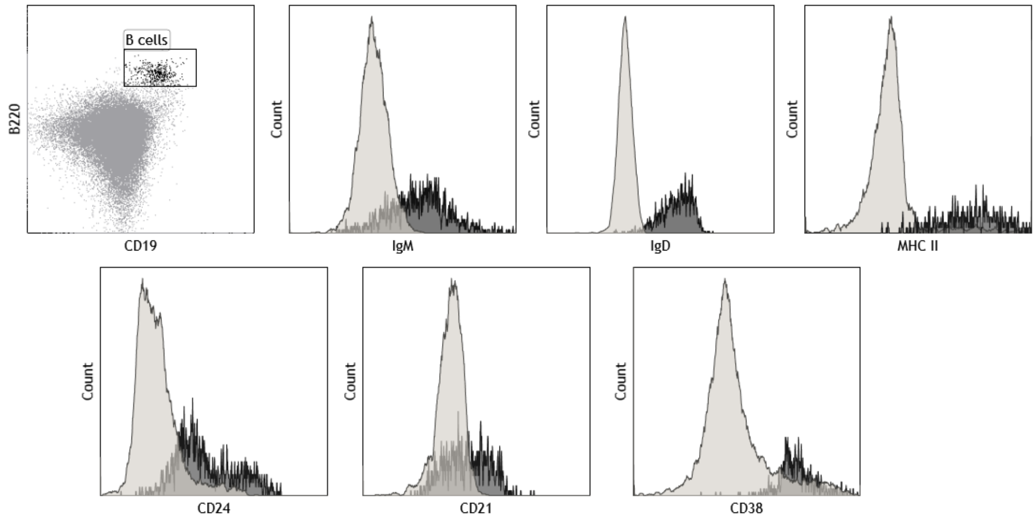

Supplementary Figure 4. Representative flow cytometric overlays of day 5.5 pc uterine B cells (dark grey) compared to non-B cells stained with B cell receptor markers B220, CD19, IgM, IgD, differentiation markers CD24 and CD21, and activation marker CD38. Uterine B cells exhibit high expression of these phenotypic markers compared to non-B cells. Surface marker expression was verified in three independent experiments.
